# Efficacy and safety of acupuncture and moxibustion for herpes zoster: A protocol for systematic review and network meta analysis

**DOI:** 10.1097/MD.0000000000021905

**Published:** 2020-09-04

**Authors:** Na Zhang, Kun Liu, Yalin She, Weixuan Zhao, Jingchun Zeng, Guohua Lin

**Affiliations:** a Guangzhou University of Chinese Medicine; b First Affiliated Hospital of Guangdong Pharmaceutical University; c Department of Acupuncture, The First Affiliated Hospital, Guangzhou University of Chinese Medicine, Guangzhou, People's Republic of China.

**Keywords:** acupuncture, herpes zoster, network meta-analysis, protocol

## Abstract

**Introduction:**

Herpes zoster (HZ) is currently treated primarily with antiviral drugs, yet this treatment has been debated. Acupuncture is becoming a more important treatment in this protocol. For example, pain intensity is lower among HZ patients who receive acupuncture plus moxibustion than among those who receive pharmacotherapy. There are many types of acupuncture interventions, including electroacupuncture, moxibustion, bloodletting. In this study, a network meta-analysis (NMA) is used to rank various interventions of acupuncture.

**Methods and analysis:**

Electronic searches of abstracts and titles will be performed in MEDLINE, EMBASE, CENTRAL, CBM, CNKI, CQVIP, and Wanfang Data databases, from inception to December 31, 2019. Published and unpublished controlled trials with different acupuncture interventions will be selected, trials of antiviral drugs as the control group. All patients of HZ will be included, except for those diagnosed with PHN, immunocompromised patients, or those with complications. The effective therapy rate and the incidence of PHN are primary outcomes. The NMA will be analyzed with Stata 13.0 and GeMTC 0.14.3.

**Discussion:**

The NMA will be established to compare various interventions of acupuncture for the therapy of HZ, that could resolve the limitations of previous methodologies with this protocol. It will be possible to determine the best acupuncture intervention for more primary outcomes of therapy, including subgroup analysis of patients with aged ≥50 years and those of aged <50 years.

**Ethics and dissemination:**

The NMA does not require ethical approval. The data analyzed is not personal. It is only systematically used to evaluate the effectiveness of acupuncture treatments. The results will be disseminated through international conference reports and peer-reviewed manuscripts.

**Strength and limitations of this study:**

A comprehensive methodology is established to rank various interventions of acupuncture by which best evidence-based intervention may be recommended for those population groups of aged ≥50 years and aged <50 years.

**PROSPERO registration number:**

CRD42019118369.

## 1 Introduction

Herpes zoster (HZ) occurs when the varicella zoster virus invades the ganglion and skin. It is characterized by neuralgia and cluster herpes extending along the peripheral nerve.^[1–4]^ HZ mainly affects individuals aged ≥50 years, due to decreasing varicella zoster virus specific cell-mediated immunity with advancing age and it is usually self-limiting in immunocompetent patients^[5]^ with the rash resolving within 3 to 4 weeks.^[6]^ The guidelines for HZ management of the European consensus (S2k)^[7]^ indicate that HZ of any localization of patients ≥50 years of age needs the treatment of an recommended antiviral medication. Besides antiviral therapy, pain treatment and auxiliary local treatment are also clinical suggestions for HZ treatment.

Though antiviral drugs can reduce healing time, yet a recent Cochrane review^[8]^ has reported that they do not reduce the incidence of postherpetic neuralgia (PHN) which may increase additional mental and health cost. Furthermore, long-term excessive use of antiviral drugs (valacyclovir 8 g per day) may lead to hemolytic anemia, thrombocytopenia, or renal insufficiency.^[9,10]^ The 18% to 41% proportion of patients suffering from HZ may still have persistent burning and paroxysmal stimulation, even if their herpes has been cured.^[11–15]^ The chronic PHN pain not only affects their quality of life, but also interferes with emotional and social functions.^[14,16–18]^ Therefore, an effective HZ cure with minimal adverse effects is in high demand.^[19–21]^ One study has documented that acupuncture is more effective than pharmacotherapy for patients suffering from HZ.^[22]^ The ratio of the change exceeding 30% on pain reduction was 2.67:1 for the treatment and there was also a significant reduction in the incidence of PHN (1 month after rash resolution) in those who received acupuncture plus moxibustion compared to pharmacotherapy. Other outcomes may be general benefits such as time to resolution of rash, time to crust formation, time to cessation of new lesion formation, and adverse events. A systematic review (in press)^[23,24]^ assessed that wet cupping was superior to medications for the number of cured patients, the number of patients with improved symptom, and the incidence rate of PHN.

Although there are a multitude of acupuncture interventions, the clinical evidence on efficacy and safety is insufficient. Doctors need additional evidence in order to make appropriate clinical choices.

### 1.1 Objectives

The protocol provides an objective and replicable method for the extraction and analysis of data to:

1. rank the sequence of multiple interventions of acupuncture on primary outcomes to obtain the optimum intervention for HZ.
2. Establish subgroup analysis of patients aged ≥50 years and those of aged <50 years for efficacy of treatment.

## 2 Materials and methods

An network meta-analysis (NMA) is utilized to assess the effective therapy rate and the incidence of PHN with numerous acupuncture interventions as primary outcomes. Traditional meta-analyses are limited, and only used for 2-group direct comparisons, failing to generate the complete evidence picture for interventions’ efficacy. Nevertheless, current research usually employs either 3-arm interventions or 2 studies with different control groups. For instance, consider 3 interventions: fire needle A, surrounding acupuncture B, and antiviral drugs C. Only if we were to combine meta-analysis from the fire needle versus antiviral drugs (AC) and that of surrounding acupuncture versus antiviral drugs (BC), could an indirect comparison (AB) be accomplished.^[25,26]^ Consequently, an NMA^[27–29]^ that substitutes for the traditional meta-analysis will be developed.

### 2.1 Criteria for included studies

#### 2.1.1 Study design

Published and unpublished randomized controlled trials (RCTs) and randomized cross-over studies which select for HZ treatment with acupuncture will be included, irrespective of language and publication. All relevant studies will be included regardless of nationality, sex, or whether the subject is an inpatient or outpatient. Furthermore, to avoid publication bias, studies with small sample size will also be included. However, non-prospective, non-random or incorrectly randomized clinical research literature, reviews, experimental reports, and clinical case reports will be excluded.

### 2.2 Participants

Any patients who suffer from HZ will be included. However, patients diagnosed with PHN,^[22]^ as well as those with severe complications such as Ramsay Hunt syndrome or zoster opthalmicus, will be excluded. Moreover, some special patients, such as those with auto-immune diseases or pregnant women, will also be excluded. Age, sex, initial VAS and other baseline characteristics will not be restricted.

### 2.3 Interventions

All acupuncture-based HZ treatments, including electroacupuncture, moxibustion, bloodletting, cupping, fire needle, plum blossom needle, auriculo-acupuncture, and combination interventions consisting of any 2 or 3 acupuncture methods are considered interventions. Meanwhile, treatments combining herbs, antivirals or physical therapy will be excluded. The control group will be antiviral drugs, such as Acyclovir or Famciclovir.^[30]^

### 2.4 Patient and public involvement

There is not any patient and public involvement in the included studies.

### 2.5 Outcome measurements

It has been suggested that the therapeutic effective rate, and the PHN incidence at 1 month, be established as the primary outcomes. PHN is usually defined as pain that persists 4 weeks after the herpes resolves, or pain that recurs over 4 weeks after the pain has resolved. Yet, a study^[14]^ has calculated PHN incidence at the times of 1 month, 3 months, 6 months, and 12 months with acupuncture for HZ as the intervention. It concluded that there were minimal disparities observed in mean reduction of PHN incidence after 3 months. The incidence of PHN will be analyzed by 2 subgroups of age ≥50 and age <50 for that eighteen studies assessing the effects of age showed an increased risk of PHN with greater age. A study has defined therapeutic effective rate as 30% lesion improvement,^[31]^ which will also be analyzed according to the factor of age. The secondary outcomes are cutaneous symptoms, pain intensity (pain visual analog scale), adverse events (defined as harmful reactions unrelated to the therapy) and related quality of life (HRQoL), measured on the Zoster Brief Pain Inventory, Initial Zoster Impact Questionnaire, or other quality of life measures.

### 2.6 Search strategy

MEDLINE, EMBASE, CENTRAL, CBM, CNKI, CQVIP, and Wanfang databases will be searched from inception to December 31, 2019. The search terms used will be intervention (acupuncture and moxibustion), disease (HZ, zoster, varicella zoster virus, variants, and shingles), study design (RCT, comparative studies, controlled, placebo), irrespective of language and publication. Missing data for unpublished studies and grey literature will need to be collected by phone or email to the author. Preferred reporting items for systematic review and meta-analysis pictures for the NMA^[26,32]^ will also need to be produced before publication. Medical Subject Heading terms: acupuncture, and moxibustion can be connected with “or.” The terms: acupuncture, HZ, and RCT can be connected with “and.” The search strategy of all relevant databases is detailed in Figure 1 .

**Figure 1:**
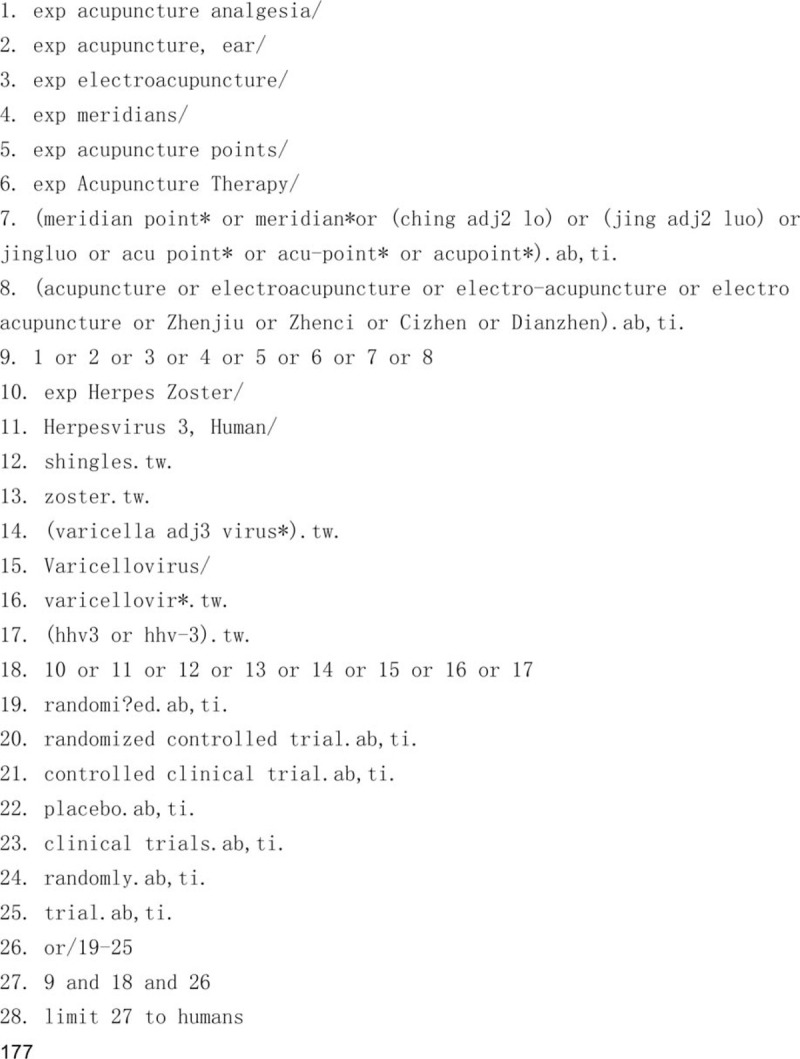
Search strategy.

**Figure 1 (Continued):**
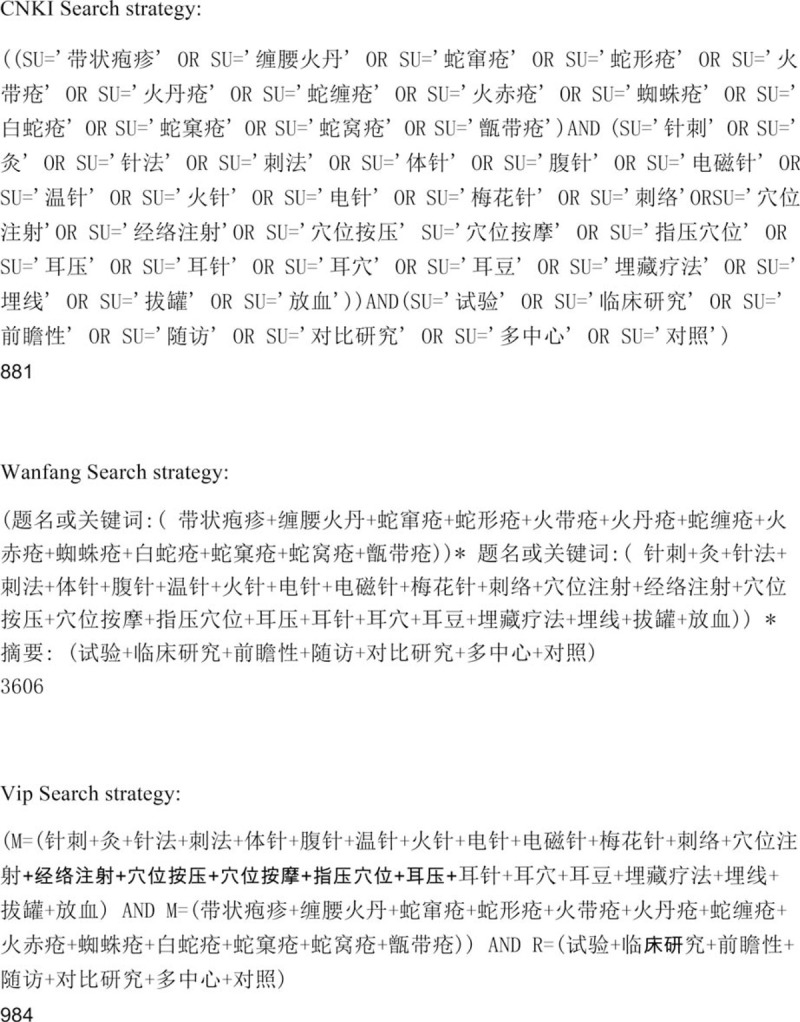
Search strategy.

### 2.7 Study identification and selection

**Figure 2:**
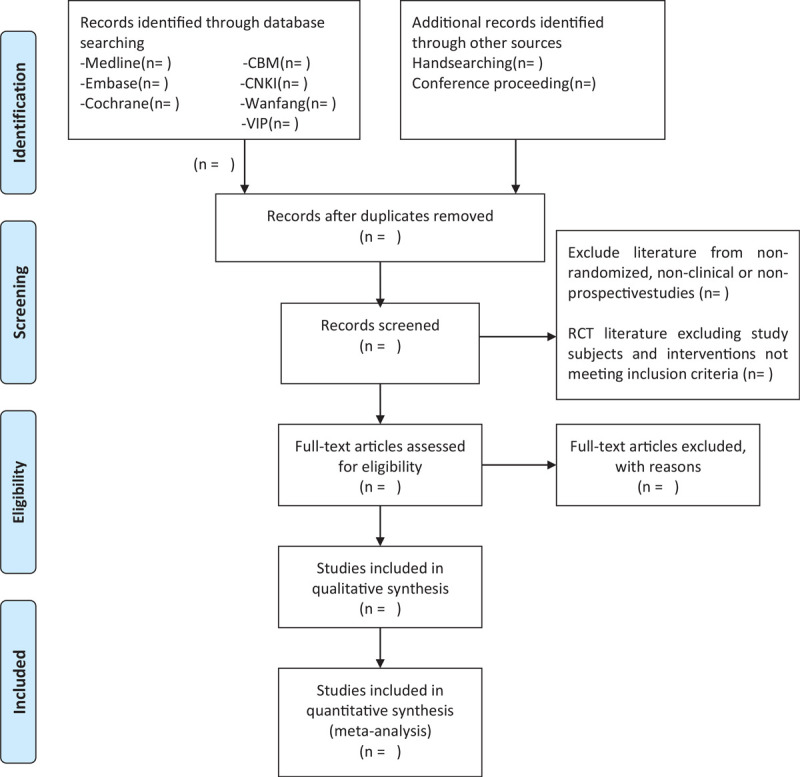
The PRISMA flow chart. PRISMA = preferred reporting items for systematic review and meta-analysis.

To begin, the titles and abstracts of the qualified studies will be searched by both electronic and manual methods; Duplicate studies will by identified by searching the same ID^[33]^ in various clinical study databases or other unpublished websites. Duplicates will be deleted with Endnote X9. Then, 2 independent staff members who are familiar with the literature management tool will finalize the 50 studies’ screening trainings. Next, the consistency of the 2 authors’ measurements when making simple inclusion/exclusion decisions will be calculated with Kappa statistics. A Kappa 0.40 to 0.59 will indicate good consistency, and 0.60 to 0.74 will indicate a better result. Anything 0.75 or above is ideal.^[34]^ Lastly, 2 researchers (NZ, KL) will independently check the abstract, scrutinize the full texts, scan the relevant literature and decide whether the study should be included according to the criteria, that will be applied (Fig. 2). Even if the study is excluded, the reasons will be explained in the table. Any disagreement will be resolved by the third researcher (WZ).

### 2.8 Data extraction and management

The literature search record management will be managed by EndNote X9. Two extraction forms will be produced for the critical results. One will be used to extract the signature of the RCT including research Plan ID/any identification ID,^[33]^ publication time, publication language, author's nationality, number of authors, nature of author's work unit (ie, hospital or university), number of author work units, funding, type of acupuncture intervention, sample size, and informed consent information. The other extraction form will also contain the essential information: owner of the study, sex, age, intervention measures (frequency, descriptions, durations), therapy time, and outcomes. One reviewer will enter these data as variables into the database, which will be cross-checked independently by another reviewer. They will consult with an additional reviewer (YS) on variables that lack necessary information.

### 2.9 Bias risk

NZ and a second reviewer will independently validate the domains with the revised Cochrane Collaboration’ tool RoB2.^[26,35]^ This will entail random sequence generation, allocation concealment, blinding of participants and personnel, incomplete outcome data, selective outcome reporting, and other bias, to summarize the results of the RCTs’ quality and the risk of bias according to 3 criteria. This is reported as high risk of bias “−,” low risk of bias “+,” or unclear risk of bias “?.” Any disagreement in assessment will be resolved with the suggestions of a third reviewer. Finally, the effects of studies with high risk of bias in the overall domain will be analyzed by sensitivity analyses.

### 2.10 Direct comparison and NMAs

We need to first construct the network map, owning to the majority of studies being either 3-arm studies or diverse interventions consisting of 2 studies. This is done with Stata V.16 (Stat Corp LLC). This can accomplish traditional pairwise meta-analysis between each direct comparison.

However, indirect comparisons which could be transformed into the direct comparison with the same control group also need to be analyzed with the NMA. By contrast, with diversified acupuncture interventions, all NMA outcomes are illustrated using GeMTC V.1.4.3, (MRC Biostatistics Unit, Cambridge, UK). This includes the effective therapy rate and the PHN incidence, pain intensity, and adverse effects. The therapeutic effective rate is expressed as ORs for dichotomous outcomes with 95% credible intervals (CrI). However, the continuous data will be expressed as means and SDs with the studies’ respective 95% CrIs. Both fixed- and random-effects models will be run for the above outcomes.^[36,37]^ When the deviance information criterion exceeds 5 in the Bayesian framework, the differences between the 2 models will be deemed significant. Depending on the level of heterogeneity, either the consistency model or the inconsistency model will be selected. The random effects model will be applied only if there are obvious differences.^[36]^ Moreover, *X*^2^ and *P* can depict whether there is any heterogeneity; the degree of heterogeneity is assessed by the *I*^2^ statistic. If *I*^2^ > 50%, the statistical heterogeneity is high.^[38]^

Lastly, an NMA will also be published with the graphics contribution plot, a comparisons adjusted funnel plot, and surface under the cumulative ranking curve^[39–42]^ graphs. The weight of each node in the network plot, consisting of nodes and edges, will be proportional to the corresponding acupuncture interventions for each outcome. A comparison-adjusted funnel plot will be conducted to assess the potential publication bias for all included studies (if more than 10 studies are present). The results of the acupuncture interventions will be ranked by the surface under the cumulative ranking curve, along with its 95% CrI and a rank-heat plot.^[26,39,43]^

### 2.11 Analysis by subgroup

Subgroup analysis will be performed based on the population characteristic of age that can modify results for the different outcomes.

## 3 Discussion

HZ is correlated with age and pressure, the most common clinical symptoms of which are clustered blisters and pain. The patients aged ≥50 years are adapt of developing the PHN because of poor immune function. However, acupuncture appears to be a critical intervention for treatments with vital outcomes of effective therapy rate and decrease the incidence of PHN comparing with antiviral medication. This study protocol compares several acupuncture interventions with NMA to select the best method for drafting clinical guidelines.

A recent study^[44]^ of intervention with fire needle in HZ treatment contrasted with pharmacotherapy has reported that both PHN incidence and the VAS score were lower. There have also been other meta-analyses of treatments published in China on relevant interventions for traditional Chinese medicine,^[45]^ acupuncture,^[46]^ moxibustion methods.^[4]^ Surprisingly, many published reports have shown that acupuncture interventions are more effective than comparable Western medicine. At present, no research ranks acupuncture interventions and determine the optimal acupuncture method. Additionally, no research has used the incidence of PHN as a predominant outcome. This may be because PHN is often considered a complication of HZ, and thus overlooked. With more attention to patient quality of life and subjective feelings, both the effective therapy rate outcomes and the PHN incidence need to be assessed in studies with direct and indirect comparisons of acupuncture interventions. Only in this way, can the optimal intervention for HZ treatment be achieved.

Moreover, the difference of therapy between acupuncture and antiviral medication of the sub-group of age ≥50 may be more significant than that of the group of age <50 with the best intervention of acupuncture.

In sum, the NMA will be crucial to ranking diversified acupuncture interventions for patients suffering from HZ. This will determine the optimal intervention, which will provide valuable evidence for clinical treatment. It is well known that once this finding is included in clinical guidelines, it will afford clinicians and patients with optimum acupuncture intervention, especially with various ages.

## 4 Limitations

The study with this methodology has some limitations. For example, missing data which may have come from gray literature or other unpublished full texts may have affected the number of RCTs included. If any vital literature on acupuncture interventions were to have been left out, the final rankings of interventions for HZ therapy would have been skewed.

## Author contributions

**Conceptualization:** Jingchun Zeng.

**Data curation:** Na Zhang.

**Supervision:** Jingchun Zeng.

**Validation:** Yalin She.

## References

[1] You Jeong K Chang Nam L Chi-Yeon L Population-based study of the epidemiology of herpes zoster in Korea. J Korean Med Sci 2014;29:1706 10. 25469074 10.3346/jkms.2014.29.12.1706 PMC4248595

[2] Nimmrich S Horneff G Incidence of herpes zoster infections in juvenile idiopathic arthritis patients. Rheumatol Int 2015;35:465 70. 25583050 10.1007/s00296-014-3197-6

[3] Shum D Arenas CG Disseminated herpes zoster. J Am Osteopath Assoc 2015;115:175. 25722364 10.7556/jaoa.2015.034

[4] Su-Ying W Wen-Liang L Epidemiology of pediatric herpes zoster after varicella infection: a population-based study. Pediatrics 2015;135:e565. 25713285 10.1542/peds.2013-4037

[5] Antonio V Gerd G Jana H Current management of herpes zoster: the European view. Am J Clin Dermatol 2005;6:317 25. 16252931 10.2165/00128071-200506050-00005

[6] Daniel AC Melody VS Stephen KT Treatment of varicella-zoster virus and postherpetic neuralgia. Dermatol Ther 2000;13:258 68.

[7] Werner RN Nikkels AF Marinović B European consensus-based (S2k) guideline on the management of herpes zoster - guided by the European Dermatology Forum (EDF) in cooperation with the European Academy of Dermatology and Venereology (EADV), part 2: treatment. J Eur Acad Dermatol Venereol 2017;31:20 9. 27579792 10.1111/jdv.13957

[8] Chen N Li Q Yang J Antiviral treatment for preventing postherpetic neuralgia. Cochrane Database Syst Rev 2014;CD006866. 24500927 10.1002/14651858.CD006866.pub3 PMC10583132

[9] Y.Z C.Z Observation on the therapeutic effect of Xuefu Zhuyu Decoction on postherpetic neuralgia. Chin J Clin 2007;35:60 1.

[10] M W. Xuefu Zhuyu Decoction in the treatment of postherpetic neuralgia. Zhejiang J Integr Tradit Chin West Med 2012;22:891 2.

[11] Robert HD Russell KP Pain and its persistence in herpes zoster. Pain 1996;67:241 51. 8951917 10.1016/0304-3959(96)03122-3

[12] R. Edgar HS The nature of herpes zoster: a long-term study and a new hypothesis. Proc R Soc Med 1965;58:1 20. 14267505 10.1177/003591576505800106 PMC1898279

[13] Johnson RW The future of predictors, prevention, and therapy in postherpetic neuralgia. Neurology 1995;45: Suppl 8: S70 2. 8545029 10.1212/wnl.45.12_suppl_8.s70

[14] Tamara U Monica T Lamberto M Acupuncture for the treatment of severe acute pain in Herpes Zoster: results of a nested, open-label, randomized trial in the VZV pain study. BMC Complement Altern Med 2011;11:1 8. 21639941 10.1186/1472-6882-11-46 PMC3125389

[15] Watson CP Oaklander AL Postherpetic neuralgia. Pain Pract 2002;2:295 307. 17156037 10.1046/j.1533-2500.2002.02039.x

[16] Robert HD John WG Anne Louise O Diagnosis and assessment of pain associated with herpes zoster and postherpetic neuralgia. J Pain 2008;9: 1 Suppl 1: S37 44. 18166464 10.1016/j.jpain.2007.10.008

[17] Robert HD Richard W O’Connor AB Healthcare costs of acute and chronic pain associated with a diagnosis of Herpes Zoster. J Am Geriatr Soc 2007;55:1168 75. 17661954 10.1111/j.1532-5415.2007.01231.x

[18] Jennifer K Edith MC Robert RW Acute pain in Herpes Zoster and its impact on health-related quality of life. Clin Infect Dis 2004;39:343 8. 10.1086/421942 15307000

[19] Meghan WM Mullane KM Stanley LL Herpes zoster and radiation therapy: what radiation oncologists need to know about diagnosing, preventing, and treating herpes zoster. Pract Radiat Oncol 2014;4:58 64. 24621425 10.1016/j.prro.2013.02.004

[20] Sophie G Pierre T Hana S Incidence of Herpes Zoster in HIV-infected adults in the combined antiretroviral therapy era: results from the FHDH-ANRS CO4 cohort. Clin Infect Dis 2015;60:1269 77. 25601456 10.1093/cid/ciu1161

[21] Winthrop K Yamanaka H Valdez H Herpes Zoster and tofacitinib therapy in patients with rheumatoid arthritis. Arthritis Rheumatol 2014;66:2675 84. 24943354 10.1002/art.38745 PMC4285807

[22] Meaghan EC L.H Kaiyi W Acupuncture plus moxibustion for herpes zoster: a systematic review and meta-analysis of randomized controlled trials. Dermatol Ther 2017;1 7. 10.1111/dth.12468 28338265

[23] Huijuan C Chenjun Z Jianping L Wet cupping therapy for treatment of herpes zoster: a systematic review of randomized controlled trials. Altern Ther Health Med 2010;16:48 54. PMC3151529 21280462

[24] Huijuan C Mei H Xun L Clinical research evidence of cupping therapy in China: a systematic literature review. BMC Complement Altern Med 2010;10:70. 21078197 10.1186/1472-6882-10-70 PMC3000376

[25] Andrea C Julian PTH John RG Conceptual and technical challenges in network meta-analysis. Ann Intern Med 2013;159:130 7. 23856683 10.7326/0003-4819-159-2-201307160-00008

[26] Renata Giacomini Oliveira Ferreira L Luísa Rocco B Julia Simões Corrêa G Effectiveness of non-pharmacological strategies in the management of type 2 diabetes in primary care: a protocol for a systematic review and network meta-analysis. BMJ Open 2020;10:e034481. 10.1136/bmjopen-2019-034481 PMC7045081 31932394

[27] Joseph Elmunzer B Amit GS Jeremy BS Comparing the effectiveness of competing tests for reducing colorectal cancer mortality: a network meta-analysis. Gastrointest Endosc 2015;81:700 9.e3. 25708757 10.1016/j.gie.2014.10.033 PMC4766592

[28] Clary JF Gordon HG Frcpc K Which surgical treatment for open tibial shaft fractures results in the fewest reoperations? A network meta-analysis. Clin Orthop Relat Res 2015;473:2179 92. 25724836 10.1007/s11999-015-4224-y PMC4457757

[29] Schwendicke F Jäger AM Paris S Treating pit-and-fissure caries: a systematic review and network meta-analysis. J Dent Res 2015;94:522 33. 25710951 10.1177/0022034515571184

[30] Björn P Anders H Use of acyclovir, valacyclovir, and famciclovir in the first trimester of pregnancy and the risk of birth defects. JAMA 2010;304:859 66. 20736469 10.1001/jama.2010.1206

[31] Nanjing University Press, State Administration of Traditional Chinese Medicine of the People's Republic of China. Standards for Diagnosis of Diseases and the Evaluation of Therapeutic Effects in Chinese Medicine. Vol 101. 1994;76–80.

[32] Moher D Liberati A Tetzlaff J Preferred reporting items for systematic reviews and meta-analyses: the PRISMA statement. BMJ 2009;339:b2535. 19622551 10.1136/bmj.b2535 PMC2714657

[33] Lars J Peter CG Tom J Index of the human papillomavirus (HPV) vaccine industry clinical study programmes and non-industry funded studies: a necessary basis to address reporting bias in a systematic review. Syst Rev 2018;7:1 0. 29347995 10.1186/s13643-018-0675-z PMC5774129

[34] Orwin RG Cooper H Hedges LV Evaluating coding decisions. Russell Sage Foundation, The Handbook of Research Synthesis. New York (NY): 1994.

[35] Lars J Asger S P-M David R TL Evaluation of the Cochrane tool for assessing risk of bias in randomized clinical trials: overview of published comments and analysis of user practice in Cochrane and non-Cochrane reviews. Syst Rev 2016;5:80. 27160280 10.1186/s13643-016-0259-8 PMC4862216

[36] Dias S Welton NJ Caldwell DM Checking consistency in mixed treatment comparison meta-analysis. Stat Med 2010;29:932 44. 20213715 10.1002/sim.3767

[37] Xiaofang T Qiancheng H Ye C Optimal dose-fractionation schedule of palliative radiotherapy for patients with bone metastases: a protocol for systematic review and network meta-analysis. BMJ Open 2020;10:e033120. 10.1136/bmjopen-2019-033120 PMC6955492 31911518

[38] Mao L Jian C Changzhi L Cytochrome CYP2C19 polymorphism and risk of adverse clinical events in clopidogrel-treated patients: a meta-analysis based on 23,035 subjects. Arch Cardiovasc Dis 2013;106:517 27. 24080325 10.1016/j.acvd.2013.06.055

[39] Georgia S Ades AE Ioannidis JP Graphical methods and numerical summaries for presenting results from multiple-treatment meta-analysis: an overview and tutorial. J Clin Epidemiol 2011;64:163 71. 20688472 10.1016/j.jclinepi.2010.03.016

[40] Ludovic T Adeline A Philippe R Impact of reporting bias in network meta-analysis of antidepressant placebo-controlled trials. PLoS One 2012;7:e35219. 22536359 10.1371/journal.pone.0035219 PMC3335054

[41] Ludovic T Nassima A Aïda B Uncertainty in treatment rankings: reanalysis of network meta-analyses of randomized trials. Ann Intern Med 2016;164:666 73. 27089537 10.7326/M15-2521

[42] Jeroen PJ Thomas T Joseph CC Indirect treatment comparison/network meta-analysis study questionnaire to assess relevance and credibility to inform health care decision making: an ISPOR-AMCP-NPC good practice task force report. Value Health 2014;17:157 73. 24636374 10.1016/j.jval.2014.01.004

[43] Areti Angeliki V Sharon ES Alexandros F The rank-heat plot is a novel way to present the results from a network meta-analysis including multiple outcomes. J Clin Epidemiol 2016;76:193 9. 26939929 10.1016/j.jclinepi.2016.02.016

[44] J-x W W-x Z J-c Z Systematic review and sequential analysis on treatment of herpes zoster pain mainly by fire needle therapy. Acupunct Res 2019;44:677 85. 10.13702/j.1000-0607.190004 31532139

[45] Guangzhou University of Chinese Medicine, Y.C Literature Review on the Treatment of Herpes Zoster with Traditional Chinese. 2009.

[46] Qiao D Meta analysis of the efficacy of acupuncture and drug therapy for postherpetic neuralgia. J Liaoning Univ Tradit Chin Med 2017;19:179 82.

